# Land use / land cover map of Vavatenina region (Madagascar) produced by object-based analysis of very high spatial resolution satellite images and geospatial reference data.

**DOI:** 10.1016/j.dib.2022.108517

**Published:** 2022-08-05

**Authors:** Camille Lelong, Hasina Herimandimby

**Affiliations:** a CIRAD, UMR TETIS, F- 34093 Montpellier, France; b Consultant, Lot VT1Ter VQSG Andohaniato-Ambohipo, Antananarivo 101, Madagascar

**Keywords:** Remote sensing, Pleiades, OBIA, Spatial database, Landscape, Agroforestry, Analanjirofo, Madagascar

## Abstract

Hereby presented data consists of a land use / land cover map of an 84 km^2^ part of the Vavatenina district, in the Analanjirofo Region of the east coast of Madagascar, where the landscape is dominated by woody vegetation. This map was obtained by processing very high spatial resolution multispectral images acquired by the Pleiades satellite sensor in 2018. Pleiades data were produced by Airbus defence & Space (France), and distributed by the French National Space Agency (CNES, France). An object-based approach was chosen to exploit the advantages of such images, allowing the use of various texture indices derived from the image to discriminate between different surfaces sharing similar radiometric properties while being structurally different. The object-based image analysis consists of iterative segmentations of the image into homogeneous regions, called objects, which can afterwards be classified using radiometric and textural variables. The reference database, computed out of field knowledge and used to train the mapping methodology, is also described here. It includes 2105 georeferenced points labelled with one of the 9 following classes: Built up/road/bare areas, Annual crops/pasture/short vegetation, Clove dominated park, Clove monoculture, Diversified agroforest, Diversified park, Plantation of woody species, Shrubby fallow, Woody fallow. It is formatted as a geographical information system, accessible for any other purpose like further methodology development. These ground-truth data also helped to the definition of the 13 thematic land use / land cover types to be distinguished in the area. Multiple segmentation levels were thus necessary to gradually derive these classes, with different classification algorithms depending on the level. The final map provides an exhaustive description of the area with a good overall accuracy, reaching 80%. It is openly available as an ESRI shapefile to allow quantitative spatial analysis in key applications such as agriculture, environment, or food security.

## Specifications Table

**Table:** 

Subject	Computer Science (Computer Science Applications, Information Systems, Signal processing)
Specific subject area	Remote sensing signal processing. Image analysis & classification. Mapping. GIS useful for landscape ecology and sustainable agriculture.
Type of data	Vector data (ESRI shapefile) Geographical Information System
How the data were acquired	Satellite VHSR imagery data were acquired by the Pleiades sensor in September 2018. They were produced by Airbus defence & Space, France, and distributed by the French National Space Agency (CNES), France, at special fees for scientists under the ISIS program. These data were delivered in orthorectified mode. The textural variables and vegetation indices appended to the image as additional features were derived using the L3Harris’ ENVI® software. Ground-truth data included in the spatial reference database consisted of 422 GPS waypoints, acquired during the winter season of 2019 with a GARMIN eTrex® 20 GPS. An infrared false-colour composition of the Pleiades image was loaded as the base-map into the GPS to support the exploration and land-use class definition. The interpretation of the land use / land cover type was directly made in the field, along with additional observations that could be useful for the image processing, but are not included in the database. Additional photo-interpreted reference points (1683) were selected in the satellite image multiband compositions based on expert knowledge reinforced by field initial survey. The object-based image analysis (OBIA) was performed under Trimble's eCognition® software, and consisted in iterative segmentations followed by hierarchical classifications of the features derived from the satellite image. Classification algorithms were chosen depending on the level, including expertized thresholding and nearest neighbour classifications. The classification accuracy assessment, and the production of the two final databases as shapefiles were performed using ESRI's ArcGIS®, and QGIS platforms.
Data format	Raw data (shapefile, ESRI)
Description of data collection	To produce the reference database, surveyed fields were chosen to encompass the diversity of the studied area landscape, and to be homogeneously distributed over the area. They also had to be representative of their class. Finally, the waypoint was recorded close to the center of the field.
Data source location	The data cover an area of 84 km^2^, including parts of the territory of 3 communes (Vavatenina, Ambohibe, Anjahambe) of the Vavatenina District, in the Analanjirofo Region of northeastern Madagascar. The frame of the satellite image used for the map has the following geographical boundary (in decimal degrees): 17.4209°S to 17.5003°S in latitude, and 49.1047° to 49.1962°E in longitude. All the points included in the reference database fit into this frame.
Data accessibility	(1) H. Herimandimby and C. Lelong, 2022. “Ground-truth reference spatial database for Vavatenina District, Analanjirofo Region, Madagascar, 2019”. CIRAD Dataverse, V1. Repository name: CIRAD Dataverse, V1 Data identification number: 10.18167/DVN1/XJCXCS Direct URL: https://doi.org/10.18167/DVN1/XJCXCS (2) C. Lelong and H. Herimandimby, 2022, "Very high spatial resolution land use / land cover map of an 84 km^2^ part of the Vavatenina District, Analanjirofo Region, Madagascar, 2019”, CIRAD Dataverse, V1 Repository name: CIRAD Dataverse, V1 Data identification number: 10.18167/DVN1/KDNOV4 Direct URL: https://doi.org/10.18167/DVN1/KDNOV4

## Value of the Data

• The produced map provides precise, objective, and quantitative information about proportions and spatial organisations of key land cover types such as cash-crop-based agroforestry systems.
• This map can support research in tropical landscape ecology and agriculture monitoring at the local scale, and it can foster territorial development planners seeking to improve cash-crop-based value chains in the mapped area.
• The developed mapping methodology can be reproduced by remote sensing specialists on other similar satellite data sets, to map complex and dense tropical environments.
• Ground-truth reference database can be reused by remote sensing specialists to assess different classification methods, or to perform multitemporal processing allowing change detection and landscape dynamics studies.

## 1 Data Description

The data presented in this article are of two kinds: (1) a reference database, and (2) a land-use/land-cover map. Both products concern an area covering 84 km^2^, located in the Vavatenina district, Analanjirofo Region, on the north-eastern coast of Madagascar, and both are provided as a geographical information system (GIS) in ESRI's shapefile format, in the standard UTM local projection: WGS 84 UTM 39 South, EPSG code 32,739. Both datasets are referenced in the CIRAD Dataverse and freely available for download [dataset] [1] [dataset] [2].

**Fig. 1:**
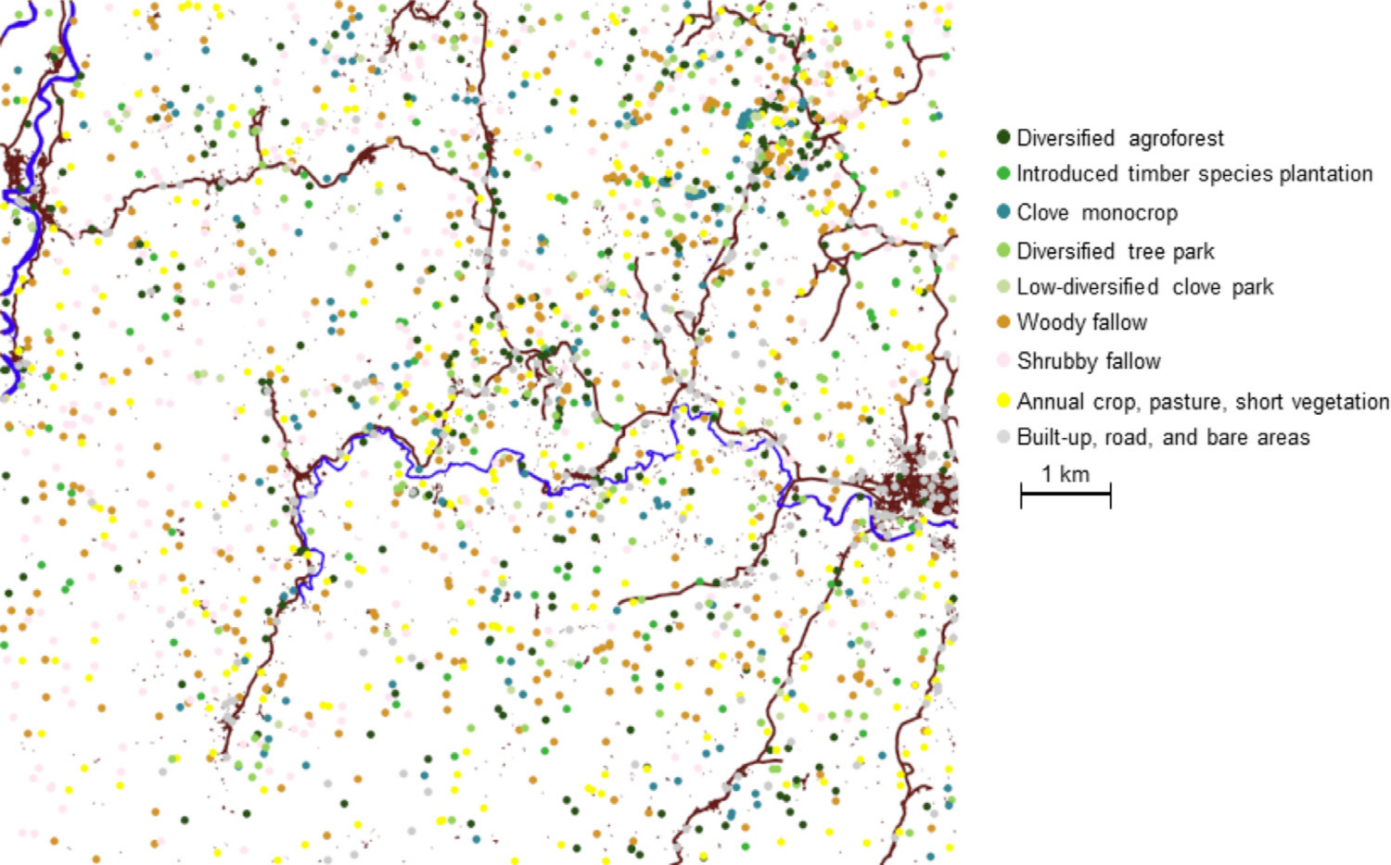
Spatial distribution of the georeferenced points included in the reference data base, displayed as coloured dots depending on their type (doi:10.18167/DVN1/XJCXCS). Blue and brown lines, respectively correspond to rivers, and to roads and towns, as recorded in OpenStreetMap (https://www.openstreetmap.org).
(2) The land-use/land-cover map is derived from the processing of a very high spatial resolution satellite image acquired by Pleiades sensor in September 2018. This GIS contains polygons, each one corresponding to a homogeneous piece of land in regards to the land-use/land-cover types nomenclature defined for this region, and described at Table 2. These quite precise classes correspond to deeper interpretation of the 9 initial survey classes (cf. Table 1), based on observations made during the field work and not included into the database. The first attribute of the shapefile stores the label of the class recognized for this piece of land, listed in the Table 2, while the second attribute indicates its corresponding area expressed in hectares. The overall precision of this classification reaches 80%. This GIS can easily be displayed in any dedicated software to produce a map, thus giving an exhaustive spatial representation of the lands in the studied area, at the spatial resolution of 0.5 m/pixel (cf. Fig. 2).

(1) The reference database GIS includes 2105 points, representative of the diversity of lands in the area. A first column of the attribute table of this shapefile indicates literally, for each point, his class label corresponding to one of the 9 types of land-use or land-cover present in the area, and listed in Table 1. These points were documented thanks to field surveys: 422 ground-truth points, or by further photo-interpretation of the satellite image, based on expert knowledge: 1683 additional points. A second column of the shapefile informs about the origin of this labelling, i.e. “GT” for direct ground-truth observation and “PI” for photointerpretation. These control points are distributed between the different types depending on the diversity and the proportion of area covered by each type, as described at Table 1. Their spatial distribution is quite homogeneous throughout the area, as it is represented at Fig. 1.

**Table 1:** Initial land use / land cover type names registered for the georeferenced points included in the reference database, and number of points recorded for type.

Class name	Number of points included in the database	Label in final nomenclature
Diversified agroforest	317	1
Introduced timber species plantation	102	2
Clove monocrop	196	3
Diversified tree park	143	4
Low-diversified clove park	70	5
Woody fallow	445	6 + 7
Shrubby fallow	305	8 + 9
Annual crop, pasture, short vegetation	303	10
Built up, road, and bare areas	224	11

**Table 2:** Land use / land cover map nomenclature description (13 classes).

Class label	Class name	Class description
1	Diversified agroforest	High-density vegetation mixing cultivated and non-cultivated trees of significant height and diameter, shrubs, and herbaceous vegetation (cropped or not)
2	Introduced timber species plantation	High-density woody vegetation dominated by introduced timber species as *Acacia mangium, Grevillea* sp. or *Eucalyptus* sp.
3	Clove monocrop	Mature clove trees in dense plantation, with a closed canopy where interspacing is invisible
4	Diversified tree park	Land with several woody species (clove trees, fruit trees, timber trees) in low density with interstitial herbaceous vegetation (cropped or not)
5	Low-diversified clove park	Land with clove trees in low density with poorly diversified interstitial herbaceous vegetation (cropped or not)
6	Low-diversified woody fallow	Land of uncropped shrubby and woody vegetation, characterized by a low canopy and poorly diversified (e.g. *Bambusa* sp.).
7	Diversified woody fallow	Land of uncropped shrubby and woody vegetation, characterized by a low canopy and highly diversified (e.g. Ravenala madascarensis, Grevillea sp, Bambusa sp., Litsea glutinosa, Psidium cattleianum)
8	Bramble shrubby fallow	Land of uncropped shrubby and herbaceous vegetation, poorly diversified and strongly invaded by *Rubus alceifolius* (also called giant bramble) or by bamboo
9	Diversified shrubby fallow	Land of uncropped shrubby and herbaceous vegetation, highly diversified and including for example *Ravenala madascarensis, Bambusa* sp, *Aframomum angustifolium, Imperata cylindrica,* and various ferns
10	Annual crop, pasture, short vegetation	Any short or herbaceous vegetation, either natural or cropped
11	Built up area, road, other bare soil	Any artificial or concrete surface, any road or path, any bare soil even if natural
12	Rice field (paddy)	Land dedicated to rice crop
13	Open water	Rivers, ponds, and any other water surface

**Fig. 2:**
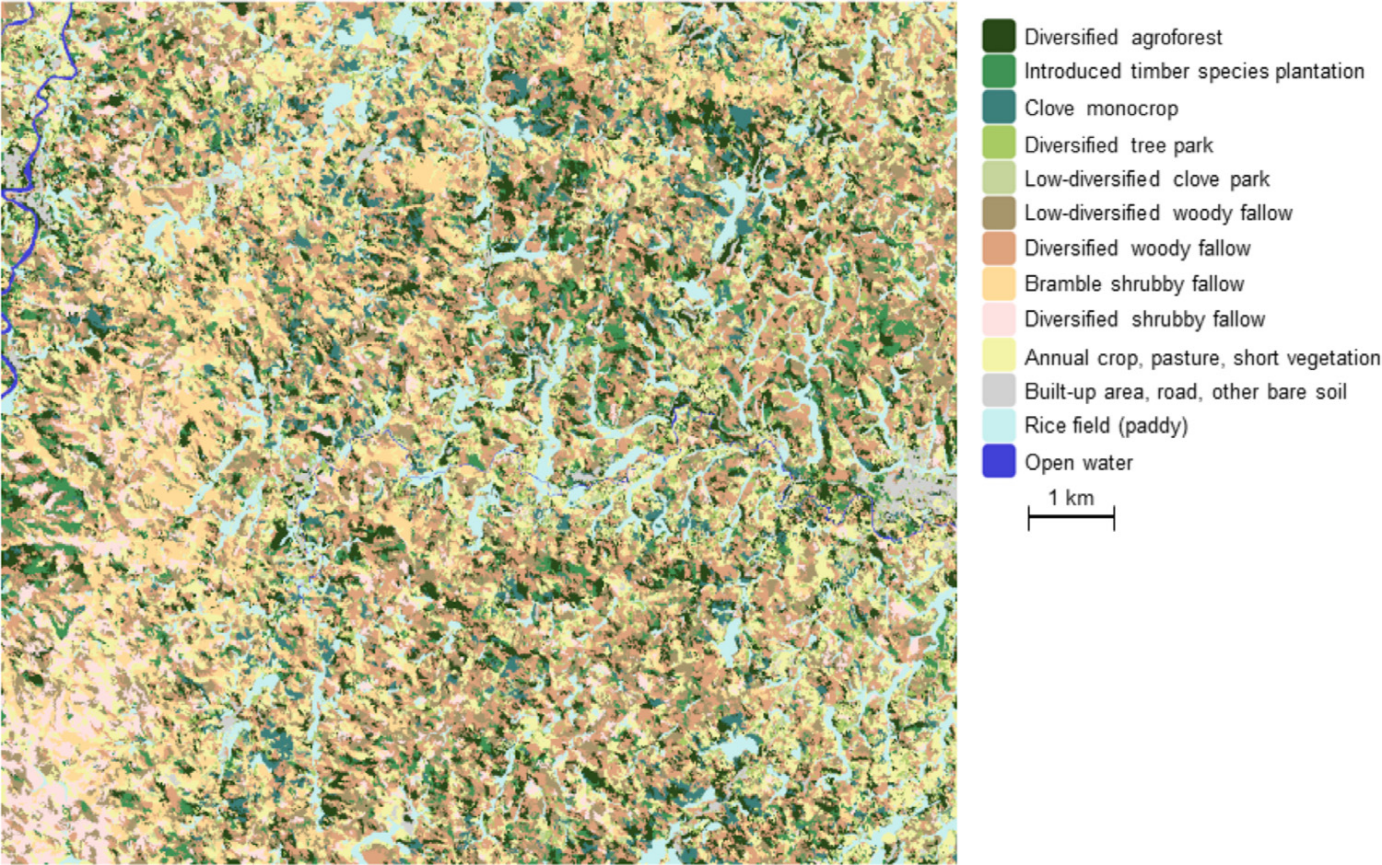
Produced land use / land cover map of the western vicinity of Vavatenina, in Analanjirofo Region of northeastern Madagascar (doi:10.18167/DVN1/KDNOV4).

## 2 Experimental Design, Materials and Methods

### 2.1 Materials

The production of the presented map relies on remote sensing data, consisting of multispectral images acquired by the Pleiades sensor (©CNES 2018, ©Airbus DS 2018) in September 2018, with less than 5% cloud cover. The frame of this satellite image covers 84 km^2^, from 17.4209°S to 17.5003°S in latitude, and 49.1047° to 49.1962°E in longitude. These data consist in a tile of 4 spectral bands, covering the ranges 430–550 nm (Blue), 490–610 nm (Green), 600–720 nm (Red), and 750–950 nm (Near Infrared, or NIR), at 2 m spatial resolution, associated with 1 panchromatic band (480–830 nm) at 0.5 m spatial resolution. They were delivered at the pre-processing level 1B, including radiometric and terrain corrected data. Thus, they were already orthorectified, that means already corrected from the topography effects, with a dedicated digital terrain elevation owned by Airbus. These data thus provide radiometric information that reveals the land surface composition and vegetation density at very small scale.

### 2.2 Methods

(a) Additional image-features layer-stacking

The studied area presents a quite complex and heterogeneous landscape, with very diverse land-covers. Most of the land is dominated by woody vegetation, some of them being cropped in diverse agricultural mixtures and practices, including in large proportions clove-based systems. Therefore, trying to discriminate these land types with only four spectral bands radiometric information is quite problematical. But clove trees, as well as others, can be regularly organized in grid, or randomly distributed within the plot, with all possible densities and levels of mixing complexity. And this structural and geometrical characteristic can be quantified using image texture metrics, thus adding a complementary level of information to the radiometric values of the pixels. Texture indices based on the grey-level cooccurrence matrix [3] are statistics derived from the organization of pairs of pixels inside a sliding window running throughout a whole single-band image, with several parameters to be fixed during the experiment (e.g., the sliding window size). We thus computed a wide range of textural indices out of the panchromatic, NIR, and red bands, which seemed to provide the higher contrast between structuring elements, and with different sliding window sizes corresponding to the scales of the main structuring elements. Then, we selected 9 of them only, by visual appreciation of the gain in information actually provided by these new features. The details about these nine indices can be found in Table 3.

**Table 3:** List of the 9 textural indices derived from the satellite images. Refer to [3] for textural indice formulae.

Name of the additional feature	Input band	Nature of the texture index	Size of the sliding window
TMean9		Mean	9 × 9 pixels = 4.5 x 4.5 m
TContr13	Panchromatic	Contrast	13 × 13 pixels = 6.5 x 6.5 m
TCorrel13		Correlation	
TEntrop13		Entropy	
TVar13		Variance	
TMean31		Mean	31 × 31 pixels = 15.5 x 15.5 m
TSecMom31		Second moment	

TMean3NIR	NIR	Mean	3 × 3 pixels = 6 x 6 m
TMean3red	Red	Mean	

Also, the well-known Normalized Difference Vegetation Index, or NDVI [4], dedicated to discriminate non-vegetated (roads, buildings…) from vegetated areas, and to distinguish different levels of vegetation density, was calculated as an additional feature. The NDVI involves the red and NIR bands using the following formula: $NDVI=\;\frac{NIR-Red}{NIR+Red}$.

Finally, before stacking together all the 15 original and additional layers, they were all recoded to 16bits and resampled at 0.5 m/pixel spatial resolution, similar to the panchromatic band.

(b) Object based image analysis (OBIA)

The high radiometric variability of pixels belonging to a single land-cover type in VHSR data, and the wide heterogeneity of the attended classes make object-based approaches [5] more suitable for their classification. The local context of each individual pixel (also called pixel neighbourhood) is considered for the identification of homogeneous regions within the image. These regions, also called objects, are delimitated through a segmentation algorithm that considers pixel values as well as geometric parameters such as scales and object compactness. In areas as complex as our studied region, with not trivial nomenclature including heterogeneous classes, a multiresolution segmentation is required to access to the discrimination of elements of different scales [6]. It results in a multi-level algorithm with successive hierarchical segmentation/classification combinations.

We carried out the whole processing using Trimble's eCognition® software. Three segmentation levels were then required, with a specific set of parameters for each one: the “shape”, the “compactness” and the “scale” factors, that have to be tuned to provide the best segmentation performance in relation with the considered level. To define the proper value for each parameter at each level, a first arbitrary value was set: 0.5 for the shape factor to give equal weight to radiometry and shape, 0.5 for the compactness factor to let the object shape loose (compact/linear, indented/smooth, …), and 400 for the scale factor because we knew by experience that this is quite close to the optimum value for small agricultural plots in VHRS imagery. Then, these values were adjusted by successive dichotomous trials, through an experimental fine-tuning based on a visual evaluation of the resulting object segmentation. The criteria retained for this evaluation are the consistency and accuracy of the delimitation of homogeneous pieces of land that could be discriminated at the given level. The finally selected parameters are presented in the Table 4.

**Table 4:** Segmentation parameters used in eCognition® at each processing level, and weights assigned to each input layer.

Segmentation level	1	2	3
Shape factor	0.5	0.66	0.5
Compactness factor	0.5	1	0.5
Scale factor	400	535	400

Input layers and layer weight	TMean3red: 1 NDVI: 6	Green: 1 TMean31: 1 TMean3NIR: 3	TMean3red: 1 NDVI: 6

Corresponding land scale	0.01 ha	0.4–0.7 ha	0.1 ha

**Fig. 3:**
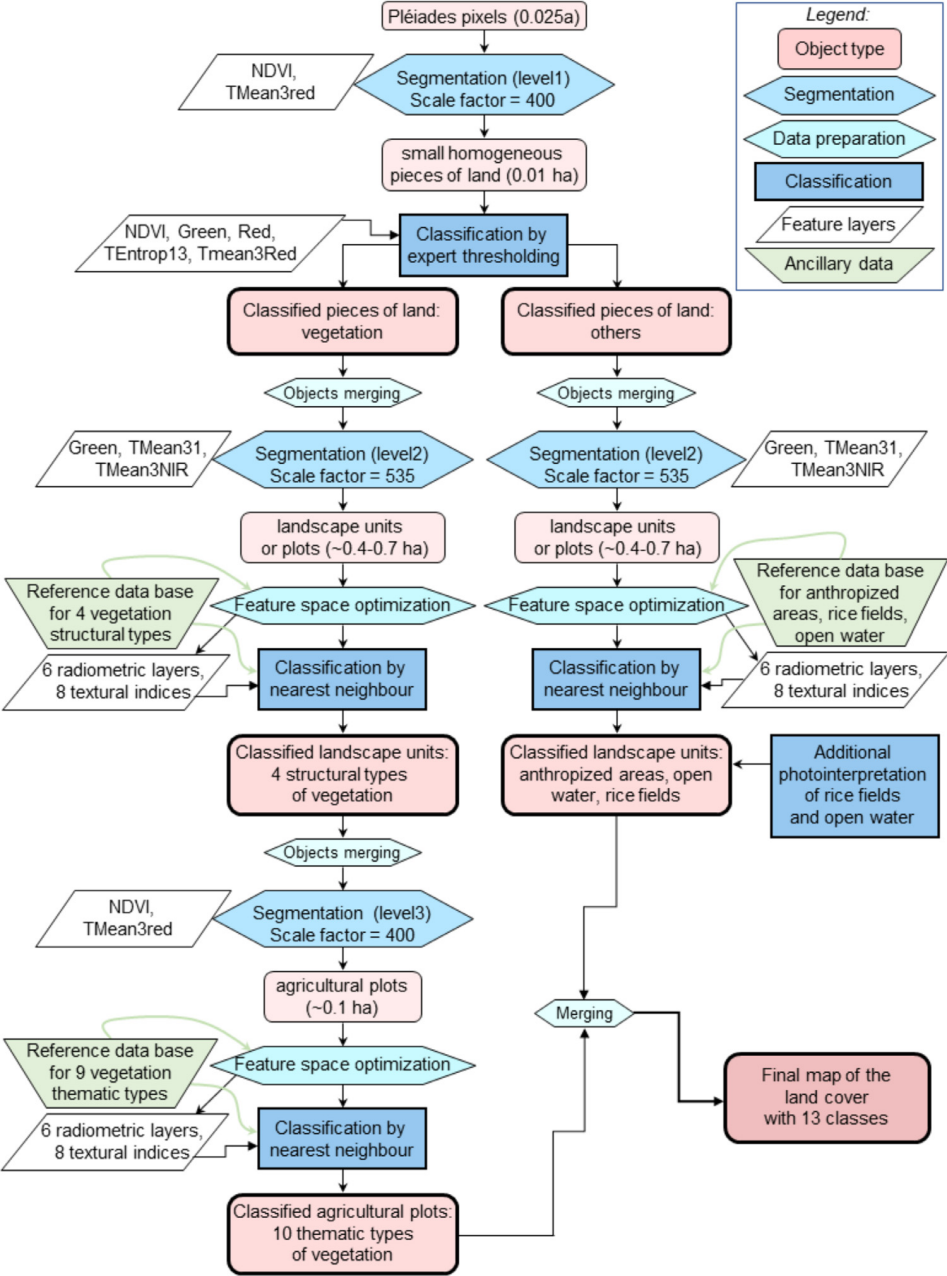
OBIA classification process implemented for the land use / land cover map production.

The feature layers used as input for the homogeneity calculation have also be determined at each level, along with the weight given to each layer. This setting was done empirically too, considering the observed homogeneity of known pieces of lands in regards to the desired classes. The whole OBIA processing chain is described in the form of a workflow chart at Fig. 3.

Level 1:

The first level of segmentation consisted of separating the vegetated areas from non-vegetated ones: bare soils, built up areas, open water and paddy fields. The initial shape, compactness and scale factors appeared to be the most relevant at this level, resulting in small homogeneous objects (about 0.01 ha) in regards with their vegetation density (use of the NDVI) and distribution (use of the texture index Tmean3red). Experimental expert tuning was then performed to establish thresholds able to discard the non-vegetated objects.

(NDVI < 5900 and TEntrop13 < 11,502.3) or (NDVI < 5900 and Green > 450 and Red > 528 and TMean3Red > 4810).

Objects not satisfying this expression were classified as vegetation. Then, the contiguous objects belonging to the same class were merged.

Level 2:

The second segmentation was performed only on the vegetation objects obtained at level 1 and was dedicated to distinguish different density distributions in the vegetation cover. As objects at this level should be compact, to figure out large fields and encompass a kind of textural pattern, the compactness had to be the highest, i.e., set to 1, and the best shape factor finally found to be 0.66 to boost the influence of the shape in the segmentation. The scale factor had to be increased to 535 to segment objects that are more or less the same size than landscape units on the field for most land use types (about 0.4 to 0.7 ha). More precisely, we defined 4 vegetated land cover classes.

(1) Dense vegetation A, dominated by trees with large crown dimensions.
(2) Dense vegetation B, with a fuzzy canopy.
(3) Sparse vegetation, with visible tree crowns.
(4) Other vegetation, rather shrubby-typed.

We tested the three more common supervised classifiers to select the most efficient for this purpose: Nearest Neighbour (NN [7]), Random Forest (RF [8]), and Support Vector Machine (SVM [9]). A supervised classification consists in training the classification model over a set of sample objects of known class, then the model classifies all the remaining objects of the image. The stacked image layers that were considered for these tests of classification were selected using the feature space optimization tool available in eCognition® [10], leading to the rejection of only the Tmean9. So, 14 image layers were considered during the classification process. The training was based on the 422 reference points collected in the fields. Assessment of the best algorithm was achieved by comparing confusion matrices and visual observations. NN classifier thus proved to be the most reliable for our application and its results were kept for further processing. Resulting objects were also merged whenever contiguous and belonging to the same class.

In parallel, we classified the non-vegetated objects into bare soil, built up areas, rice fields, and open water surfaces using NN supervised by the samples belonging to the ground-truth database. Finally, additional photointerpretation of rice fields, guided by field references and expert knowledge, and based on several color compositions of the stacked layers, consolidated and completed this step.

Level 3:

The third segmentation aimed at precising the vegetation classes, interpreting them into the 10 more thematic types that fits the attended nomenclature (cf. Table 2), like the different agroforestry systems and tree crops that are in practice in the region. All the objects remaining unclassified at the second level were also considered for adequate classification. The segmentation was applied on the sub-objects merged at the second level, with the same parameters than the first level, but resulting in larger patches (the size of an agricultural plot) because applied on objects instead of pixels. The classification was performed again by a NN trained on the reference ground-truth points and applied to the whole scene.

(c) Validation

The 1683 photo-interpreted reference points were used evaluate the accuracy of the semi-automatic classification. They were selected randomly throughout the entire mapped area, keeping only the ones included in objects larger than 1 ha. The two first segmentation levels were visually validated throughout the processing software. Then, the confusion matrix was derived (cf. Table 5), based on the comparison between the actual class which the point belongs to, and the class determined at the end of the third level of the mapping process. Global accuracy of The OBIA classification thus provides a global accuracy of 75% and a kappa coefficient of 0.71.

**Table 5:** Confusion matrix of the OBIA classification. Class labels correspond to the initial ground-truth nomenclature defined at Table 1.

Class label	1	2	3	4	5	6 + 7	8 + 9	10	11	Total	User accuracy
1	154	7	3	5	1	19	4	0	0	193	80%
2	16	52	1	1	0	10	0	1	0	81	64%
3	34	12	99	1	3	15	2	0	0	166	60%
4	6	0	6	69	7	24	9	11	0	132	52%
5	7	1	6	32	44	25	7	3	0	125	35%
6 + 7	15	7	3	15	3	255	43	7	0	348	73%
8 + 9	1	0	0	0	0	8	170	13	0	192	89%
10	0	1	0	5	1	1	15	223	0	246	91%
11	0	0	0	0	0	0	0	0	200	200	100%
Total	233	80	118	128	59	357	250	258	200	1683	

Producer accuracy	66%	65%	84%	54%	75%	71%	68%	86%	100%		

User accuracies of most of the classes are quite good, assuring a good reliability of the map, especially considering the main land cover types. Going farther into the woody land uses, strong confusion between the different parklands arise, depending on their tree species composition. These classes are still challenging in remote sensing, because of the large complexity of mixed species canopies, and our results still range in the good published ones. For instance, the class “Low-diversified clove park” presents only 35% of user accuracy, with 25% of the validation samples being classified as “Diversified tree park”, and 20% being classified as “Woody fallows”. These errors are mainly due to the fact that these environments are composed of quite the same kind of canopies with only some differences of structure and compositional diversity, and that tree species are not discriminable from one to another. As an example, clove trees are not always distinguished from other big trees, either by shape or radiometry. The limit between one kind of tree park and another kind is also fuzzy because the level of diversity is somehow subjective. So, the choice will be up to the user to privilege typology precision over classification reliability, or reverse. To improve these classes discrimination, additional ground-truth information would be required, in order to better define the class contours. Also, deeper analysis could be conducted on the textural patterns and consequent additional features (indices) to add more structural constraints in the classification process. Herbaceous vegetation (cropped and uncropped) and non-vegetated mapped areas are totally reliable with, respectively 91 and 100% user accuracy. Rice fields and water surfaces, because of the high precision of expert photointerpretation, can also be considered as exact. OpenStreetMap data (https://www.openstreetmap.org) also helped to further validate the rivers delimitation, showing that they are even more completely mapped thanks to our work.

Finally, considering the 13 classes of the total nomenclature (Table 2), the produced map reaches more than 80% of global accuracy.

## Ethic Statement

The authors declare that the hereby presented data and data article fully comply with the Journal's policy in terms of authors’ duties, data integrity, and experimental requirements.

## CRediT authorship contribution statement

**Camille Lelong:** Conceptualization, Supervision, Resources, Investigation, Validation, Writing – review & editing. **Hasina Herimandimby:** Methodology, Resources, Investigation, Formal analysis, Validation, Data curation, Writing – original draft.

## Declaration of Competing Interest

The authors declare that they have no known competing financial interests or personal relationships that could have appeared to influence the work reported in this paper.

## Data Availability

Ground-truth reference spatial database for Vavatenina District, Analanjirofo Region, Madagascar, 2019 (Original data) (Dataverse). Very high spatial resolution land use / land cover map of an 84km^2^ part of the Vavatenina District, Analanjirofo Region, Madagascar, 2019 (Original data) (Dataverse).

## References

[1] Lelong C., Herimandimby H. (2022). Very high spatial resolution land use /land cover map of an 84km^2^ part of the Vavatenina District, Analanjirofo Region, Madagascar, 2019. CIRAD Dataverse.

[2] Herimandimby H., Lelong C. (2022). Ground-truth reference spatial database for Vavatenina District, Analanjirofo Region, Madagascar –2019, reference geospatial database. CIRAD Dataverse.

[3] Haralick R.M., Shanmugam K., Dinstein I.H. (1973). Textural features for image classification. IEEE Trans. Syst. Man Cybern..

[4] Tucker C.J. (1979). Red and photographic infrared linear combinations for monitoring vegetation. Remote Sens. Environ..

[5] Blaschke T. (2010). Object based image analysis for remote sensing. ISPRS J. Photogramm. Remote Sens..

[6] Baatz M., Schäpe A., Strobl J., Blaschke T., Griesebner G. (2002). Angewandte Geographische Informations-Verarbeitung, XII.

[7] Franco-Lopez H., Bauer M. (2001). Estimation and mapping of forest stand density, volume, and cover type using the k-nearest neighbors’ method. Remote Sens. Environ..

[8] Breitman L. (2001). Random forests. Mach. Lear..

[9] Pal M., Mather P.M. (2005). Support vector machines for classification in remote sensing. Int. J. Remote Sens..

[10] Kucharczyk M., Hay G.J., Ghaffarian S., Hugenholtz C.H. (2020). Geographic object-based image analysis: a primer and future directions. Remote Sens..

